# Quantitative analysis of the resting-state EEG power spectrum in patients with epilepsy comorbid with anxiety and depression

**DOI:** 10.1186/s42494-025-00206-6

**Published:** 2025-03-31

**Authors:** Hongxing Chen, Juan Yang, Bo Zhang, Lijia Zhang, Jing Wang, Haiqing Zhang, Hongwei Zhang, Changyin Yu, Jun Zhang, Zucai Xu

**Affiliations:** 1 Department of Neurology, Affiliated Hospital of Zunyi Medical University, Zunyi, 563003 China; 2 Key Laboratory of Brain Science, Zunyi Medical University, Zunyi, 563099 China; 3 Guizhou Key Laboratory of Anesthesia and Organ Protection, Zunyi Medical University, Zunyi, 563099 China; 4 Department of Prevention and Health Care, Affiliated Hospital of Zunyi Medical University, Zunyi, 563003 China; 5 Department of Neurology, Yinjiang Autonomous County People’s Hospital, Zunyi, 555200 China; 6 Department of Neurology, People’s Hospital of Qianxinan Prefecture, Qianxinan, 562400 China

**Keywords:** Epilepsy, Comorbidity, Anxiety, Depression, EEG power spectrum

## Abstract

**Background:**

Epilepsy is one of the common clinical disorders with comorbid anxiety and depression that severely affects their quality of life and increases their suicidality, while screening for anxiety and depression currently lacks objective identifiers. This study aimed to analyze the characteristics of the electroencephalogram (EEG) power spectrum in patients with epilepsy with comorbid anxiety and depression, utilizing resting EEG data.

**Methods:**

Resting EEG data were collected under standard conditions from two groups: patients with epilepsy comorbid with anxiety and depression (*n* = 42) and patients without comorbidities (*n* = 45). EEG power was calculated using data processing with EEGLAB and MATLAB. This study compared the absolute and relative powers of the δ, θ, α, β, and γ frequency bands, as well as the values of (δ + θ)/(α + β), between the two groups. Additionally, the correlation between the EEG power of each frequency band and anxiety and depression scores was analyzed.

**Results:**

1) Among individuals with epilepsy comorbid with anxiety and depression, lower absolute power of δ, α, and θ at specific sites was observed (*P* < 0.05), along with lower relative power of θ at certain sites (*P* < 0.05). Conversely, higher relative power of β and γ at specific sites was noted in those with comorbidities (*P* < 0.05). 2) There was no statistically significant difference in the values of (δ + θ)/(α + β) between the two groups (*P* > 0.05). 3) Depression scores exhibited a negative correlation with θ absolute power at the T3 and T4 sites (*P* < 0.05), while showing a positive correlation with β relative power at the C4 and T6 sites (*P* < 0.05). Anxiety scores displayed a positive correlation with β relative power at the F4, C3, C4 and T6 sites and γ relative power at F8 site (*P* < 0.05).

**Conclusions:**

The findings suggest that comorbid anxiety and depression may impact resting EEG power spectra in individuals with epilepsy, particularly in regions exhibiting altered network connectivity. Furthermore, a positive correlation was observed between anxiety and depression scores and β relative power in the right central and right posterior temporal regions, indicating potential screening utility.

**Supplementary Information:**

The online version contains supplementary material available at 10.1186/s42494-025-00206-6.

## Key points

The study compared EEG power spectra in epilepsy patients with and without comorbid anxiety and depression, Focusing on δ, θ, α, β, and γ bands.

It unveiled that in epilepsy patients with combined anxiety and depression, slow wave (δ, θ) and α power values in some brain regions decreased (*P*<0.05), while the power values of fast wave (β, γ) increased (*P*<0.05). These changes are mainly concentrated in the frontal and temporal lobes.

This study also revealed that increased β power in specific brain regions correlates with higher anxiety and depression scores, suggesting its potential for screening purposes.

## Background

Epilepsy is a chronic neurological disorder characterized by abnormal synchronous discharges in the brain, and it ranks among the most prevalent clinical conditions. Anxiety and depression are recognized as prevalent psychological comorbidities in people with epilepsy (PWE) [1, 2]. These comorbidities substantially impact the quality of life of PWE and elevate their risk of suicide.

Electroencephalography (EEG) is the simplest tool to evaluate brain activity, which reflects the integrated electrophysiological activity of a group of neurons and contains a wealth of physiological and psychological information. Resting-state EEG is recorded while the subject is awake, with eyes closed, relaxed, and not engaged in any specific task. It reflects baseline brain activity and intrinsic network connectivity in the absence of external task interference and is more closely related to spontaneous brain activity and default network activity. It is used to assess resting-state functional connectivity of the brain and to diagnose neuropsychiatric disorders such as epilepsy and depression. In contrast, non-resting-state EEGs are recorded while individuals perform specific cognitive, sensory, or motor tasks. This type of EEG reflects the activity state of the brain when processing external information and performing tasks, and it is related to the response of the brain to specific stimuli and the performance of cognitive functions. It is used to assess the activation state and functional connectivity of the brain, as well as to monitor the response of the brain to treatment. Overall, resting-state EEGs provide information about baseline brain activity in the task-free state, whereas non-resting-state EEGs provide insights into brain activity during the performance of a specific task. Therefore, resting-state EEGs were used in this study.

In recent years, related studies have demonstrated that PWE exhibit varying levels of background EEG activity, which extend beyond epileptic discharge regions to encompass alterations in brain activity affecting the entire brain. This is characterized by an elevated interictal EEG power spectrum across the δ, θ, β and γ rhythms [3]. In addition, anxiety and depression may also influence the EEG background waves. To the best of our knowledge, no studies have been undertaken to examine alterations in the resting EEG power spectrum among patients with epilepsy who also have comorbid anxiety and depression, and the underlying mechanisms of these changes remain poorly understood.

Quantitative electroencephalography (QEEG) measurements provide insights into the fundamental instability of cortical arousal, which is frequently observed in various anxiety disorders [4, 5]. One study revealed greater power in the β1 and β2 bands in patients with anxiety and depression compared to a control group, with statistically significant differences in β1 power in the parietal and occipital lobes, as well as β2 power in the frontal lobe [6]. Furthermore, several studies have documented EEG abnormalities in patients exhibiting mild depressive symptoms, with increased θ power in the right anterior central gyrus and elevated α power in the right superior temporal gyrus observed among patients with mild depression [7, 8].

QEEG uses computers to calculate and visualize EEG activity in both the time and frequency domains, encompassing recording digital EEG signals that are subsequently processed, transformed, and analyzed using sophisticated mathematical algorithms [9]. Studies have shown that QEEG possesses a broad spectrum of clinical applications, including the management of neuropsychiatric disorders, epilepsy, stroke, dementia, brain trauma, posttraumatic stress disorder, and Parkinson's disease [10–14]. QEEG is considered a potential intermediate biomarker for the diagnosis of mental diseases [15]. QEEG introduces a innovative technology for extracting features from EEG signals, including specific frequency band analysis, signal complexity assessment, connectivity analysis, and network analysis [16], with power spectrum analysis serving as its core component. The power spectrum analysis of QEEG constitutes a quantitative analysis of background brainwaves, effectively illustrating the distribution and variability of brainwaves across the α, β, θ, δ, and γ bands.

Considering the chronic and persistent characteristics of mood disorder symptoms, we hypothesized that these symptoms are mediated by interictal dysfunction rather than by relatively transient neurological changes occurring during seizures. Consequently, in this study, we employed power spectrum analysis to examine spontaneous brain activity at rest in PWE who also have comorbid anxiety and depression. Should a significant and stable difference be identified between the individuals with epilepsy comorbid with anxiety and depression and those without comorbidities, based on the resting EEG power spectrum, it would have a positive impact the diagnosis and treatment of epilepsy associated with these conditions.

## Methods

### Participants

Data were collected from PWE who were monitored by video EEG at the Affiliated Hospital of Zunyi Medical University from October 1, 2020, to July 31, 2021. All participants met the diagnostic criteria outlined in the 2017 epilepsy diagnosis and treatment guidelines established by the International League Against Epilepsy [17] .

The inclusion criteria were as follows: 1) aged between 14 and 60 years; 2) capable of cooperating with the scale examination and EEG recordings; 3) all scale assessments completed within one day before or after EEG acquisition; and 4) right-handedness. The exclusion criteria included: 1) acute and chronic systemic infection; 2) long-term alcohol consumption; 3) neurological disorders (such as cerebral infarction, intracranial space-occupying lesions, brain trauma, and encephalitis); 4) cognitive impairment (defined by a Mini-Mental State Examination [MMSE] score of less than 27); 5) left-handedness or mixed handedness; 6) neuropsychiatric disorders such as schizophrenia; and 7) use of anti-anxiety, depression, or benzodiazepines.

Currently, the assessment of anxiety and depression in PWE is predominantly carried out using a range of neuropsychological scales. Nevertheless, the sensitivity of each scale is constrained, and subjectivity continues to pose a considerable challenge. Relevant studies have indicated that the Generalized Anxiety Disorder-7 (GAD-7) [18] and the Neurological Disorders Depression Inventory for Epilepsy (NDDI-E) [19] demonstrate greater sensitivity in assessing anxiety and depression within this specific population. Therefore, this study aimed to utilize the GAD-7 and NDDI-E to assess anxiety and depression status in epilepsy patients. Based on the scores from the GAD-7 and NDDI-E scores, patients with epilepsy were classified into two groups: those comorbid with anxiety and depression group (GAD-7 score > 6 and NDDI-E score > 12) and those without anxiety and depression group (GAD-7 score ≤ 6 and NDDI-E score ≤ 12).

All participants comprehended the objectives of this study and voluntarily consented to participate by providing written informed consent. This study received approval from the Ethics Committee of the Affiliated Hospital of Zunyi Medical University (KLLY-2020-115).

### Neuropsychological examination

The neuropsychological assessment scales used in this study comprised the GAD-7, NDDI-E, and MMSE.

### Electroencephalogram

A long-range NicoletOne video EEG monitoring system was utilized. Sixteen electrodes (FP1, FP2, F3, F4, F7, F8, T3, T4, T5, T6, C3, C4, P3, P4, O1, and O2) along with two reference electrodes (A1 and A2) were positioned on the scalp according to the international 10–20 system. During the recording sessions conducted in a quiet room, subjects remained awake in bed with their eyes closed and relaxed for 10 min prior to the examination. Sample data from eyes closed for 5min were selected for analysis.

### EEG processing

During the data preprocessing and analysis phase, blinding of subject groups was maintained to minimize subjective bias. Subsequently, EEGLAB and MATLAB were employed to detect and label artifacts, thereby reducing potential human judgment bias. Following automated detection, a manual review was conducted to ensure that no data were overlooked or incorrectly labeled. Five-minute resting EEG segments were randomly selected for analysis. Additionally, another EEG expert independently reviewed the data selection process.

The established standards for EEG frequency band were as follows: δ band (2–4 Hz), θ band (5–7 Hz), α band (8–12 Hz), β band (15–29 Hz), and γ band (30–46 Hz). In this study, a total of 16 electrode sites were selected for analysis, including the frontal pole (FP1, FP2), frontal region (F3, F4), temporal region (F7, F8, T3, T4, T5, T6), central region (C3, C4), parietal region (P3, P4), and occipital region (O1, O2).

The power values were derived using EEGLAB (SCCN, San Diego, USA, Version 2021.1) and MATLAB(Mathworks, Natick, MA, USA,Version R2021b) for EEG data processing. The original data were processed with a finite impulse response (FIR) bandpass filter with a frequency range of 1–46 Hz to eliminate interference, and a 10-second segment was excluded from both the beginning and end of the dataset to further mitigate any residual interference. Upon completion of the processing, the data were manually reviewed to identify and eliminate any interference factors. EEG data were recorded using bilateral earlobe reference electrodes in accordance with international standards, and the whole-brain averaged reference was used for referencing the EEG data. For each patient, four 10-second segments of interference-free data were selected, from which the absolute and relative power of each frequency band was calculated alongside the ratio of (δ + θ)/(α + β), and subsequently averaged for each patient.

### Statistical analysis

The statistical software SPSS (IBM Corp., Armonk, NY, USA, Version 29.0) was used for data processing. Categorical variable data are presented as the number of cases or composition ratios and were analyzed using the Chi-square test. When numerical variables exhibited a normal distribution, the mean ± standard deviation was used for statistical description, and the independent sample *t*-test was applied for comparison between the two groups. When the data did not conform to a normal distribution, the median (interquartile range) was used for statistical description, and the Mann–Whitney U test was used. Statistical significance was established at *P* < 0.05 (two-tailed). Spearman’s correlation analysis was conducted to examine the relationships among anxiety and depression scores and EEG power.

## Results

### Baseline data

**Fig. 1:**
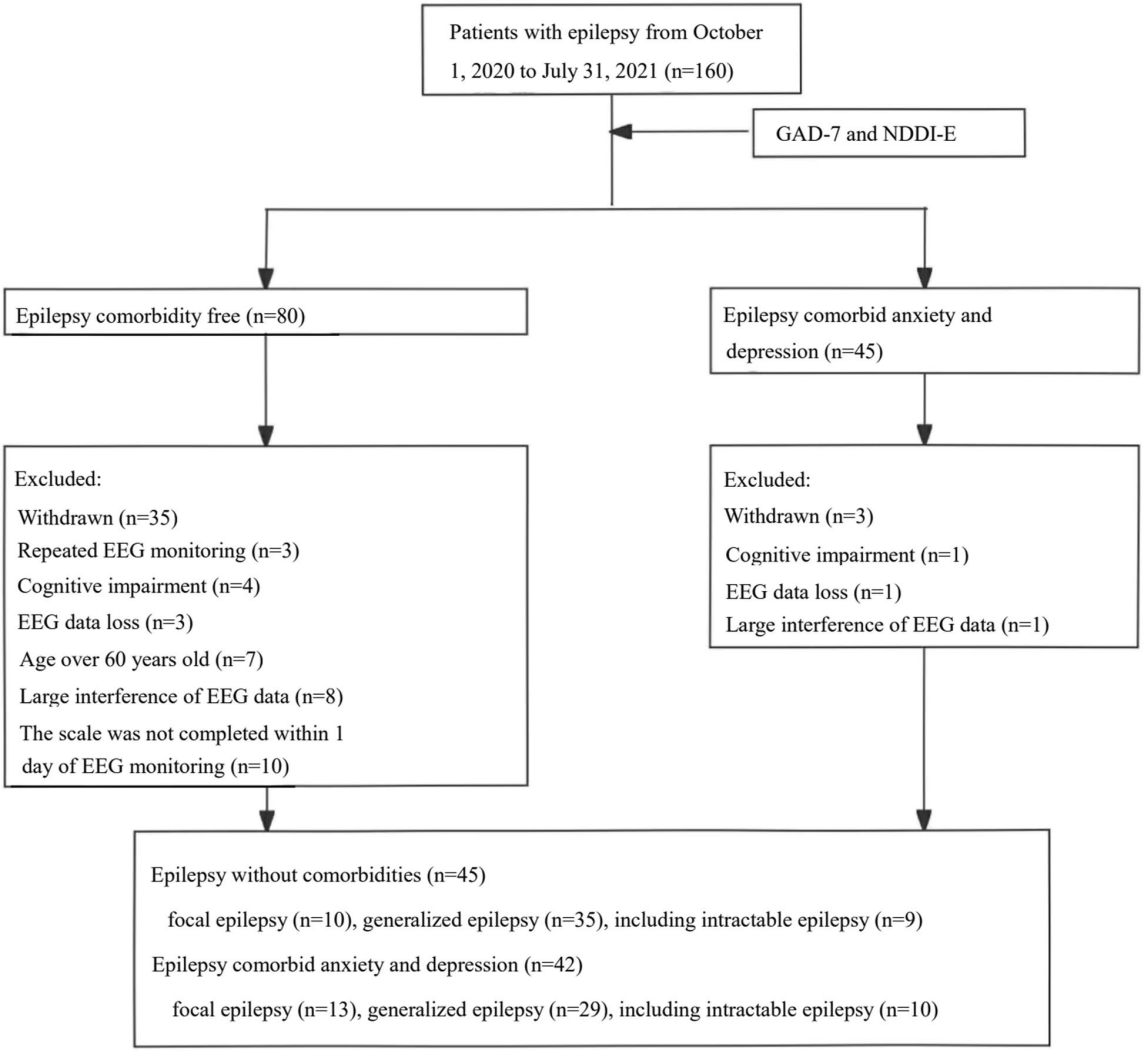
Flow chart for screening and grouping of patients with epilepsy

This study collected data from 160 epilepsy patients who underwent video-EEG monitoring at the Affiliated Hospital of Zunyi Medical University from October 1, 2020, to July 31, 2021. According to the GAD-7 and NDDI-E scores, there were 45 patients in the epilepsy group comorbid with anxiety and depression group and 80 patients in the epilepsy group without anxiety and depression. After excluding 1 patient with cognitive dysfunction, 1 patient with lost EEG data, and 1 patient with EEG data interference, a total of 42 patients were ultimately included in the epilepsy comorbid anxiety and depression group. In contrast, 45 patients were included in the epilepsy group without anxiety and depression after excluding 3 patients with repeated EEG monitoring, 4 patients with cognitive impairment, 3 patients with lost EEG data, 7 patients older than 60 years, 8 patients with EEG data interference, and 10 patients who did not complete the scale evaluation within 1 day of EEG monitoring (Fig. 1). There was no significant difference in the baseline data between the two groups (*P* > 0.05) (Table 1).

**Table 1:** Demographics and neuropsychological examination results

	Epilepsy comorbid anxiety and depression	Epilepsy comorbidity free	*P*-value
Number of cases	42	45	
Sex (male/female)	21/21	26/19	0.467
Age	30.45 ± 12.93	27.53 ± 12.07	0.279
Age of onset	23.30 ± 12.74	19.38 ± 9.99	0.113
Duration of epilepsy (months)	60 (36, 120)	36 (9, 126)	0.054
Number of hospitalizations due to epilepsy	0.5 (0, 1)	0 (0, 1)	0.088
Number of concomitant antiseizure medications	1.12 ± 0.80	1.02 ± 0.89	0.597
Take medicines regularly (yes/no)	18/24	23/22	0.441
MMSE score	28.69 ± 1.27	28.98 ± 1.12	0.269
GAD-7 score	11.50 ± 3.14	2.47 ± 1.91	0
NDDI-E score	17.38 ± 3.06	8.73 ± 2.06	0

Note: Age, age of onset, number of concomitant antiseizure medications, and scale scores are expressed in mean ± standard deviation; the duration of epilepsy and the number of hospitalizations due to epilepsy are expressed in the median (quartile); take drugs regularly (yes/no) and gender are expressed in the number of cases. independent sample *t*-test, Mann–Whitney U test and chi-square test were used, respectively

*GAD-7* Generalized Anxiety Disorder-7, *NDDI-E* Neurological Disorders Depression Inventory for Epilepsy, *MMSE* Mini-Mental State Examination

### Analysis of the resting-EEG power spectra of the two groups

#### Comparison of the absolute power of each frequency band in each brain area between the two groups

Sixteen sites were analyzed, revealing that the δ absolute power in the epilepsy with comorbid anxiety and depression group decreased by 1.6% to 31.3% compared to that of the epilepsy group without comorbidities. The difference observed in the left middle temporal region (T3) between the two groups was significant (*P* < 0.05) (see Table S1). The θ absolute power in the epilepsy group with comorbid anxiety and depression decreased by 2.8% to 41.7%, with significant differences observed in the frontal region, temporal region, and right central region (F3, F4, C4, F7, F8, T3, T4, and T6) between the two groups (*P* < 0.05) (Table 2). The α absolute power in the epilepsy group with comorbid anxiety and depression varied, decreasing or increasing by 5.5% to 50.4% compared to that of the epilepsy group without comorbidities, with a significant difference found in the left middle temporal region (T3) (*P* < 0.05) (Table S2). No significant differences were observed in β or γ absolute powers between both groups (*P* > 0.05).

**Table 2:** θ absolute power of each site (× 10^12^)

Sites	Epilepsy comorbid anxiety and depression (*n* = 42)	Epilepsy comorbidity free (*n* = 45)	*P*-value
FP1	1.65 (1.67)	2.20 (3.72)	0.096
FP2	1.71 (1.6)	2.09 (3.96)	0.169
F3	1.00 (1.89)	1.51 (2.89)	0.016*
F4	1.03 (1.92)	1.75 (3.12)	0.026*
C3	0.73 (1.35)	0.95 (1.57)	0.064
C4	0.80 (1.41)	1.05 (1.65)	0.041*
P3	0.76 (1.23)	1.08 (1.72)	0.088
P4	0.80 (1.18)	1.03 (1.67)	0.096
O1	1.85 (1.74)	1.91 (4.41)	0.285
O2	1.85 (2.55)	2.13 (3.24)	0.171
F7	0.87 (0.92)	1.33 (2.37)	0.030*
F8	0.86 (1.25)	1.42 (1.75)	0.029*
T3	0.74 (1.04)	1.27 (1.67)	0.006*
T4	0.74 (0.9)	1.17 (1.58)	0.010*
T5	1.28 (2.14)	1.73 (3.92)	0.142
T6	1.21 (1.62)	1.51 (3.81)	0.044*

Note: Data are expressed as median (interquartile range) and were statistically analyzed using the Mann–Whitney U test

^*^P value < 0.05

#### Comparison of the relative power of each frequency band in each brain area between the two groups

Sixteen sites were analyzed. Compared to the epilepsy group without comorbidities, the θ relative powers in the epilepsy group with comorbid anxiety and depression decreased or increased by 1.3% to 18.4%, with significant differences in the left frontal pole and left frontal lobe (Fp1, F3, F7) between the two groups (*P* < 0.05) (Table S3). The β relative power in the epilepsy group comorbid with anxiety and depression increased by 21.6% to 74% compared to that of the group without anxiety and depression, with significant differences noted in the central region, left parietal region, left temporal region, right occipital region, and right posterior temporal region (C3, C4, P3, O2, F7, T3, T5, and T6) between the two groups (*P* < 0.05) (Table 3). The relative γ power in the epilepsy group comorbid with anxiety and depression increased by 21.4% to 101.8% compared to that of the group without comorbidities, with significant differences were found in the frontal region, central region, left parietal region, and left temporal region (F3, F4, F7, F8, C3, C4, P3, T3, and T5) between these two groups (*P* < 0.05) (Table 4). No significant difference were observed in the δ or α relative powers between the two groups (*P* > 0.05).

**Table 3:** β relative power of each site (100%)

Sites	Epilepsy comorbid anxiety and depression (*n* = 42)	Epilepsy comorbidity free (*n* = 45)	*P*-value
Fp1	3.36 (3.33)	2.63 (2.52)	0.212
Fp2	3.57 (3.51)	2.76 (3.24)	0.405
F3	4.9 (4.48)	3.5 (3.78)	0.103
F4	4.95 (4.67)	3.55 (3.19)	0.053
C3	5.31 (4.01)	3.48 (3.36)	0.048*
C4	4.64 (4.58)	2.72 (3.2)	0.010*
P3	4.24 (4.5)	3.31 (3.64)	0.041*
P4	4.2 (4.87)	2.74 (4)	0.057
O1	4.44 (5.31)	3.65 (3.86)	0.098
O2	4.56 (5.53)	3.08 (2.99)	0.035*
F7	5.07 (3.97)	3.36 (3.92)	0.035*
F8	5.03 (4.08)	2.89 (3.74)	0.069
T3	6.43 (5.43)	3.99 (4.24)	0.014*
T4	6.35 (5.86)	3.96 (5.07)	0.080
T5	4.55 (4.43)	3.21 (3.82)	0.034*
T6	4.6 (4.51)	3.09 (3.42)	0.012*

Note: Data are expressed as median (interquartile range) and were statistically analyzed using the Mann–Whitney U test

^*^ *P* value < 0.05

**Table 4:** γ relative power of each site (100%)

Sites	Epilepsy comorbid anxiety and depression (*n* = 42)	Epilepsy comorbidity free (*n* = 45)	*P*-value
Fp1	1.1 (1.27)	0.8 (1.43)	0.308
Fp2	1.1 (1.65)	0.88 (1.42)	0.164
F3	1.34 (1.9)	0.9 (1.22)	0.045*
F4	1.2 (1.25)	0.75 (1.19)	0.040*
C3	1.02 (1.01)	0.84 (0.93)	0.040*
C4	1.13 (0.78)	0.76 (1.1)	0.043*
P3	0.92 (0.88)	0.54 (0.73)	0.037*
P4	0.88 (0.91)	0.66 (0.85)	0.060
O1	1.45 (3.1)	0.82 (2.44)	0.191
O2	1.13 (2.95)	0.56 (1.65)	0.089
F7	2.1 (1.86)	1.2 (1.64)	0.013*
F8	1.6 (2.69)	1.04 (2.36)	0.040*
T3	2.24 (2.88)	1.2 (2.98)	0.023*
T4	2.15 (3.47)	1.61 (2.61)	0.241
T5	1.14 (1.78)	0.85 (0.82)	0.024*
T6	1.16 (1.24)	0.88 (1.19)	0.085

Note: Data are expressed as median (interquartile range) and were statistically analyzed using the Mann–Whitney U test

^*^P value < 0.05

Sixteen sites were analyzed. The values of (δ + θ)/(α + β) in the epilepsy group comorbid with anxiety and depression increased or decreased by 0–27.8% compared to the comorbidity-free epilepsy group, however, the difference between the two groups were not statistically significant (*P* > 0.05).

### Correlation analysis

Anxiety scores exhibited positively correlations with the β relative power in the right frontal region, central region, and right posterior temporal region (F4, C3, C4, T6), as well as the γ relative power of the right anterior temporal region (F8). Conversely, depression scores demonstrated positive correlations with the β relative power in the right central region and right posterior temporal region (C4, T6),while exhibiting negative correlations with the θ absolute power in the middle temporal region (T3 and T4) (Table 5).

**Table 5:** Correlation between EEG power spectrum and anxiety and depression scores

β relative power			γ relative power		θ absolute power	
sites	GAD-7	NDDI-E	GAD-7	NDDI-E	GAD-7	NDDI-E
Fp1	0.123	0.047	0.091	−0.001	−0.095	−0.127
Fp2	0.091	0.026	0.112	0.036	−0.079	−0.100
F3	0.183	0.077	0.155	0.083	−0.165	−0.202
F4	0.229*	0.140	0.203	0.103	−0.159	−0.196
C3	0.230*	0.139	0.190	0.139	−0.151	−0.141
C4	0.286**	0.232*	0.178	0.149	−0.144	−0.171
P3	0.179	0.128	0.177	0.143	−0.117	−0.153
P4	0.206	0.130	0.186	0.113	−0.103	−0.110
O1	0.165	0.141	0.106	0.102	−0.011	−0.083
O2	0.210	0.164	0.170	0.137	−0.054	−0.085
F7	0.155	0.101	0.192	0.155	−0.157	−0.185
F8	0.180	0.116	0.230*	0.144	−0.187	−0.185
T3	0.198	0.123	0.157	0.131	−0.199	−0.233*
T4	0.173	0.097	0.146	0.056	−0.188	−0.211*
T5	0.181	0.160	0.204	0.201	−0.065	−0.124
T6	0.260*	0.219*	0.179	0.123	−0.117	−0.148

Note: The data in the table are correlation coefficients

^*^ *P* < 0.05

## Discussion

The energy information conveyed by brain waves across various frequency bands, including α, β, θ, δ, and γ, is reflected in the power spectrum. Previous studies analyzing interictal EEG power spectra have demonstrated that patients with idiopathic generalized epilepsy exhibit greater power across multiple frequency bands in various brain regions compared to control subjects [20–25]. Prior to myoclonic seizures, an increase in power was noted across all frequency bands, leading to speculation that elevated spectral power may indicate susceptibility to seizures [26]. Additionally, differences in EEG power spectra have been observed in depressed patients [27]. Studies have shown that depressed individuals exhibit increased resting-state α power in both anterior and posterior regions of the brain [28, 29]. However, findings from these studies vary regarding the specific frequency bands and scalp regions where such differences were found.

Herein, quantitative EEG power spectrum analysis was performed for all brain regions in each frequency band. Notably, the β relative powers of the central region, left temporoparietal region, right occipital region, and right posterior temporal region of PWE who have comorbid anxiety and depression showed significant increases. Furthermore, we observed a positive correlation between β relative power and anxiety and depression scores in some brain regions, primarily within the right central and the right posterior temporal regions. A certain correlation was identified between the β power of patients with epilepsy and comorbid depression and their depression scores [30]. Consequetnly, we hypothesize that the β relative power in both the right central and the right posterior temporal regions may be particularly sensitive to variations in anxiety and depression levels. Some researchers believe that an increase in γ power from background EEG signals may correlate negatively with epilepsy control [25]; specifically, higher γ power is associated with poorer seizure management. Our findings revealed a significant increase in γ relative power of epileptic patients with comorbid anxiety and depression in several brain regions; additionally, anxiety scores were positively correlated with γ relative power of the right anterior temporal lobe. This indicates that both anxiety and depression can elevate γ relative power in some brain regions, especially those related to temporal function. Therefore, we propose that the increased γ relative power observed in the temporal lobe predominantly results from heightened anxiety levels. The ratio of (δ + θ)/(α + β) serves as an indicator of EEG activity slowing and reflected declines in overall brain function [31]. However, our analysis did not reveal significant differences regarding this ratio between the epilepsy group comorbid with anxiety and depression and the epilepsy group without comorbidities. Our results showed that anxiety and depression do not contribute to a deceleration of background brain waves in patients with epilepsy, thus relevant EEG parameters reflecting the slowing of brain waves have limited clinical value concerning anxiety and depression.

It is well known that glutamate is an excitatory neurotransmitter, while GABA has a broad inhibitory effect, and both neurotransmitters play a crucial role in epileptic seizures. However, high concentrations of glutamate and lower cortical GABA levels have also been found in the cerebrospinal fluid and plasma of patients with major depressive disorder [32]. Our study found that the relative power values of the β and γ frequency bands were higher in epilepsy patients comorbid with anxiety and depression compared to those without comorbidities, mainly concentrated in the frontal, temporal, and central regions. The anxiety scores were positively correlated with the γ relative power values in the right anterior temporal region and the β relative power values in the right frontal, central, and right posterior temporal regions. The depression scores were positively correlated with the β frequency relative power values in the right central and right posterior temporal regions. We speculate that the increase in excitatory neurotransmitters or the reduction of GABA in epilepsy patients with comorbid anxiety and depression leads to an increase in fast wave activity, resulting in higher β and γ relative power values in the corresponding brain regions. These results suggest that the increased β and γ relative power values in the temporal region of epilepsy patients are a result of comorbid anxiety and depression.

Currently, it is believed that temporal lobe epilepsy (TLE) and psychiatric disorders share similar neural network features, including regions such as the temporal lobe, hippocampus, amygdala, frontal lobe, and subcortical structures [33, 34]. Previous studies have found that many patients with TLE and depression have reduced functional connectivity characteristics in multiple brain regions related to emotions and cognition, impaired frontotemporal network function, reduced frontal-temporal-limbic system connectivity, and changes in relative power spectra of the hippocampus, cingulate cortex, and amygdala [30, 35–38]. Ren et al. analyzed changes in neural oscillations and functional connectivity, finding reduced frontal θ oscillations and functional connectivity in patients with epilepsy and depression, confirming that changes in frontal connectivity may be a potential mechanism of depression [39]. The EEG power spectrum analysis in this study revealed a decrease in θ power among epilepsy patients with comorbid anxiety and depression, particularly in the frontal and temporal lobes, especially the left frontal lobe. The severity of depression showed a negative correlation with θ power, while the absolute power values of δ and α in the left middle temporal region were relatively low. Furthermore, patients with epilepsy who have comorbid anxiety and depression exhibited decreased slow wave power and absolute α wave power due to weakened connectivity within the frontal-temporal-limbic system.

Some studies indicate that anti-seizure medications (ASMs) affect spectral power, while others report no significant effects [40] . Both lamotrigine and sodium valproate have been shown to reduce spectral power in the δ, θ, α, and β frequency bands [41–44]. Valproate has also been reported to increase upper α power in the occipital regions, with a decrease in β power in another study [44]. A study evaluating the effects of levetiracetam in patients before and after medication initiation reported a decrease in δ and θ and an increase in α and β power [45] . Overall, evidence from the majority of studies on these three ASMs suggests that they alter spectral power in epilepsy in at least one frequency band, suggesting that the differences observed in our study may reflect mixed effects of ASMs. To exclude the influence of ASMs on our results, we grouped patients based on their medication status and compared EEG power spectrum differences between groups. We then divided both the unmedicated and medicated epilepsy groups into subgroups according to comorbid anxiety or depression status for further comparison of EEG spectra. Finally, we categorized the group with comorbid anxiety/depression alongside those without into two subgroups based on ASM use for additional analysis of EEG spectrum differences. Results are presented in Table S4.1–6, indicating that ASMs had minimal or no effect on the power spectrum.

Differences in epileptiform activity or epilepsy type may also contribute to observed power changes, even when such activity is excluded from the EEG data. Some researchers argue that the power changes in specific frequency bands are not unique to any particular disease [27]. Mental disorders are often presented as a loose set of overlapping symptoms, and other comorbidities not considered in this study may complicate our results. Therefore, analyses focusing on specific symptoms and symptom clusters could yield more detailed insights. Additionally, the EEG signal analysis method adopted here is relatively simple; it only anlyzes the EEG power spectrum, leading to a single result. However, incorporating additional indicators and adopting diverse EEG data processing methods could significantly enhance the diagnosis of epilepsy comorbid with anxiety and depression.

## Conclusions

In conclusion, this study showed that the comorbidity of anxiety and depression affects the power spectrum of resting-state electroencephalogram (REEG) in the brain regions related to PWE, particularly influencing the power values in areas with altered brain network connectivity. Epilepsy patients with comorbid anxiety and depression exhibited increased fast-wave (β, γ) power values and decreased slow-wave (θ, δ) and α power values, primarily in the frontal and temporal regions. It is hypothesized that the elevation in relative power values of fast waves (β, γ) may be linked to an increase in excitatory neurotransmitters or a decrease in GABA. The reduction in slow-wave power values (θ, δ) and α absolute power values may be associated with decreased connectivity within the frontal–temporal-limbic system. Furthermore, resting-state β relative power has clinical significance for screening anxiety and depression in epilepsy patients. Moving forward, we will analyze various EEG indices within the frequency bands of these brain regions to further investigate the EEG characteristics of comorbid anxiety and depression in epilepsy patients and improve their identification.

## Supplementary Information

Supplementary Material 1.

## Data Availability

Not applicable.

## Abbreviations

ASMs: Anti-seizure medications
EEG: Electroencephalogram
GAD-7: Generalized Anxiety Disorder-7
MMSE: Mini-Mental State Examination
NDDI-E: Neurological Disorders Depression Inventory for Epilepsy
PWE: People with epilepsy
QEEG: Quantitative electroencephalography
REEG: Resting-state electroencephalogram
TLE: Temporal lobe epilepsy

## References

[1] Kanner AM. Psychiatric comorbidities in new onset epilepsy: Should they be always investigated? Seizure. 2017;49:79–82. 28532711 10.1016/j.seizure.2017.04.007

[2] Tellez-Zenteno JF, Patten SB, Jetté N, Williams J, Wiebe S. Psychiatric comorbidity in epilepsy: a population-based analysis. Epilepsia. 2007;48(12):2336–44. 17662062 10.1111/j.1528-1167.2007.01222.x

[3] Pegg EJ, Taylor JR, Mohanraj R. Spectral power of interictal EEG in the diagnosis and prognosis of idiopathic generalized epilepsies. Epilepsy Behav. 2020;112:107427. 10.1016/j.yebeh.2020.107427 32949965

[4] Demerdzieva A, Pop-Jordanova N. Alpha asymmetry in QEEG recordings in young patients with anxiety. Prilozi. 2011;32(1):229–44. 21822191

[5] Ribas VR, Ribas RG, Nóbrega JA, da Nóbrega MV, Espécie JAA, Calafange MT, et al. Pattern of anxiety, insecurity, fear, panic and/or phobia observed by quantitative electroencephalography (QEEG). Dement Neuropsychol. 2018;12(3):264–71. 30425790 10.1590/1980-57642018dn12-030007 PMC6200158

[6] Yamada M, Kimura M, Mori T, Endo S. EEG power and coherence in presenile and senile depression. Characteristic findings related to differences between anxiety type and retardation type. Nihon Ika Daigaku zasshi. 1995;62(2):176–85. 10.1272/jnms1923.62.176 7775654

[7] Li X, Cao T, Hu B, Sun S, Li J. EEG Topography and Tomography (sLORETA) in Analysis of Abnormal Brain Region for Mild Depression. In: Ali H, Shi Y, Ascoli GA, et al., editors. Brain Informatics and Health: International Conference, BIH 2016, Proceedings. Lecture Notes in Computer Science (including subseries Lecture Notes in Artificial Intelligence and Lecture Notes in Bioinformatics). Vol 9919 LNAI. Cham: Springer International Publishing; 2016. p. 304–11. 10.1007/978-3-319-47103-7_30.

[8] Silvia S-O, Elva P-L, Maria del, Pilar P-Z. Resting EEG activity and ovarian hormones as predictors of depressive symptoms in postmenopausal women without a diagnosis of major depression. Psychology. 2012;3(9):834–40.

[9] Nuwer M. Assessment of digital EEG, quantitative EEG, and EEG brain mapping: report of the American Academy of Neurology and the American Clinical Neurophysiology Society. Neurology. 1997;49(1):277–92. 9222209 10.1212/wnl.49.1.277

[10] Fernández A, Al-Timemy AH, Ferre F, Rubio G, Escudero J. Complexity analysis of spontaneous brain activity in mood disorders: A magnetoencephalography study of bipolar disorder and major depression. Compr Psychiatry. 2018;84:112–7. 29734005 10.1016/j.comppsych.2018.03.015

[11] Geraedts VJ, Boon LI, Marinus J, Gouw AA, van Hilten JJ, Stam CJ, et al. Clinical correlates of quantitative EEG in Parkinson disease: A systematic review. Neurology. 2018;91(19):871–83. 30291182 10.1212/WNL.0000000000006473

[12] Livint Popa L, Dragos H, Pantelemon C, Verisezan Rosu O, Strilciuc S. The Role of Quantitative EEG in the Diagnosis of Neuropsychiatric Disorders. J Med Life. 2020;13(1):8–15. 32341694 10.25122/jml-2019-0085 PMC7175442

[13] Moon SY, Choi YB, Jung HK, Lee YI, Choi SH. Increased Frontal Gamma and Posterior Delta Powers as Potential Neurophysiological Correlates Differentiating Posttraumatic Stress Disorder from Anxiety Disorders. Psychiatry Investig. 2018;15(11):1087–93. 30481994 10.30773/pi.2018.09.30 PMC6259003

[14] Tedrus GM, Negreiros LM, Ballarim RS, Marques TA, Fonseca LC. Correlations Between Cognitive Aspects and Quantitative EEG in Adults With Epilepsy. Clin EEG Neurosci. 2019;50(5):348–53. 30198328 10.1177/1550059418793553

[15] McVoy M, Lytle S, Fulchiero E, Aebi ME, Adeleye O, Sajatovic M. A systematic review of quantitative EEG as a possible biomarker in child psychiatric disorders. Psychiatry Res. 2019;279:331–44. 31300243 10.1016/j.psychres.2019.07.004

[16] van Straaten EC, Stam CJ. Structure out of chaos: functional brain network analysis with EEG, MEG, and functional MRI. European neuropsychopharmacology : the journal of the European College of Neuropsychopharmacology. 2013;23(1):7–18. 23158686 10.1016/j.euroneuro.2012.10.010

[17] Scheffer IE, Berkovic S, Capovilla G, Connolly MB, French J, Guilhoto L, et al. ILAE classification of the epilepsies: Position paper of the ILAE Commission for Classification and Terminology. Epilepsia. 2017;58(4):512–21. 28276062 10.1111/epi.13709 PMC5386840

[18] Tong X, An D, McGonigal A, Park SP, Zhou D. Validation of the Generalized Anxiety Disorder-7 (GAD-7) among Chinese people with epilepsy. Epilepsy Res. 2016;120:31–6. 26709880 10.1016/j.eplepsyres.2015.11.019

[19] Tong X, An D, Lan L, Zhou X, Zhang Q, Xiao F, et al. Validation of the Chinese version of the Neurological Disorders Depression Inventory for Epilepsy (C-NDDI-E) in West China. Epilepsy Behav. 2015;47:6–10. 10.1016/j.yebeh.2015.03.012 26004785

[20] Clemens B, Szigeti G, Barta Z. EEG frequency profiles of idiopathic generalised epilepsy syndromes. Epilepsy Res. 2000;42(2–3):105–15. 11074183 10.1016/s0920-1211(00)00167-4

[21] Elshahabi A, Klamer S, Sahib AK, Lerche H, Braun C, Focke NK. Magnetoencephalography Reveals a Widespread Increase in Network Connectivity in Idiopathic/Genetic Generalized Epilepsy. PLoS ONE. 2015;10(9):e0138119. 26368933 10.1371/journal.pone.0138119 PMC4569354

[22] Niso G, Carrasco S, Gudín M, Maestú F, Del-Pozo F, Pereda E. What graph theory actually tells us about resting state interictal MEG epileptic activity. NeuroImage Clinical. 2015;8:503–15. 26106575 10.1016/j.nicl.2015.05.008 PMC4475779

[23] Santiago-Rodríguez E, Zaldívar-Uribe E. Analysis of clinical characteristics, background, and paroxysmal activity in EEG of patients with juvenile myoclonic epilepsy. Brain Sci. 2021;12(1):29. 35053773 10.3390/brainsci12010029 PMC8773902

[24] Tikka SK, Goyal N, Umesh S, Nizamie SH. Juvenile myoclonic epilepsy: Clinical characteristics, standard and quantitative electroencephalography analyses. J Pediatr Neurosci. 2013;8(2):97–103. 24082923 10.4103/1817-1745.117835 PMC3783741

[25] Willoughby JO, Fitzgibbon SP, Pope KJ, Mackenzie L, Medvedev AV, Clark CR, et al. Persistent abnormality detected in the non-ictal electroencephalogram in primary generalised epilepsy. J Neurol Neurosurg Psychiatry. 2003;74(1):51–5. 12486266 10.1136/jnnp.74.1.51 PMC1738170

[26] Sun Y, Zhang G, Zhang X, Yan X, Li L, Xu C, et al. Time-frequency analysis of intracranial EEG in patients with myoclonic seizures. Brain Res. 2016;1652:119–26. 27693884 10.1016/j.brainres.2016.09.042

[27] Newson JJ, Thiagarajan TC. EEG Frequency Bands in Psychiatric Disorders: A Review of Resting State Studies. Front Hum Neurosci. 2018;12:521. 30687041 10.3389/fnhum.2018.00521 PMC6333694

[28] Jaworska N, Blier P, Fusee W, Knott V. α Power, α asymmetry and anterior cingulate cortex activity in depressed males and females. J Psychiatr Res. 2012;46(11):1483–91. 22939462 10.1016/j.jpsychires.2012.08.003 PMC3463760

[29] Kemp AH, Griffiths K, Felmingham KL, Shankman SA, Drinkenburg W, Arns M, et al. Disorder specificity despite comorbidity: resting EEG alpha asymmetry in major depressive disorder and post-traumatic stress disorder. Biol Psychol. 2010;85(2):350–4. 20708650 10.1016/j.biopsycho.2010.08.001

[30] Scangos KW, Ahmad HS, Shafi A, Sellers KK, Dawes HE, Krystal A, et al. Pilot Study of An Intracranial Electroencephalography Biomarker of Depressive Symptoms in Epilepsy. J Neuropsychiatry Clin Neurosci. 2020;32(2):185–90. 31394989 10.1176/appi.neuropsych.19030081 PMC7429560

[31] Gawel M, Zalewska E, Szmidt-Sałkowska E, Kowalski J. The value of quantitative EEG in differential diagnosis of Alzheimer’s disease and subcortical vascular dementia. J Neurol Sci. 2009;283(1–2):127–33. 19268969 10.1016/j.jns.2009.02.332

[32] Kanner AM, Scharfman H, Jette N, Anagnostou E, Bernard C, Camfield C, et al. Epilepsy as a Network Disorder (1): What can we learn from other network disorders such as autistic spectrum disorder and mood disorders? Epilepsy Behav. 2017;77:106–13. 29107450 10.1016/j.yebeh.2017.09.014 PMC9835466

[33] Valente KD, Busatto FG. Depression and temporal lobe epilepsy represent an epiphenomenon sharing similar neural networks: clinical and brain structural evidences. Arq Neuropsiquiatr. 2013;71(3):183–90. 23563720 10.1590/s0004-282x2013000300011

[34] Vinti V, Dell’Isola GB, Tascini G, Mencaroni E, Cara GD, Striano P, et al. Temporal Lobe Epilepsy and Psychiatric Comorbidity. Front Neurol. 2021;12:775781. 34917019 10.3389/fneur.2021.775781 PMC8669948

[35] Berg AT, Altalib HH, Devinsky O. Psychiatric and behavioral comorbidities in epilepsy: A critical reappraisal. Epilepsia. 2017;58(7):1123–30. 28464309 10.1111/epi.13766 PMC5498258

[36] Chen S, Wu X, Lui S, Wu Q, Yao Z, Li Q, et al. Resting-state fMRI study of treatment-naïve temporal lobe epilepsy patients with depressive symptoms. Neuroimage. 2012;60(1):299-304. 22178816 10.1016/j.neuroimage.2011.11.092

[37] Zhang Z, Lu G, Zhong Y, Tan Q, Chen H, Liao W, et al. fMRI study of mesial temporal lobe epilepsy using amplitude of low-frequency fluctuation analysis. Hum Brain Mapp. 2010;31(12):1851–61. 20225278 10.1002/hbm.20982 PMC6870704

[38] He ZQ. Study on the characteristics of brain structure and functional connection in patients with epilepsy with depression. (In Chinese) 2018. https://www.cnki.net. Accessed 15 Jul 2024.

[39] Ren Y, Pan L, Du X, Li X, Hou Y, Bao J, et al. Theta oscillation and functional connectivity alterations related to executive control in temporal lobe epilepsy with comorbid depression. Clinical neurophysiology : official journal of the International Federation of Clinical Neurophysiology. 2020;131(7):1599–609. 32417702 10.1016/j.clinph.2020.03.038

[40] Y Hl, Helmstaedter C, Lehnertz K. Correction to: Quantitative Pharmaco-Electroencephalography in Antiepileptic Drug Research. CNS drugs. 2019;33(3):299. 10.1007/s40263-019-00603-9 PMC6424935 30806964

[41] Clemens B. Valproate decreases EEG synchronization in a use-dependent manner in idiopathic generalized epilepsy. Seizure. 2008;17(3):224–33. 17697790 10.1016/j.seizure.2007.07.005

[42] Clemens B, Piros P, Bessenyei M, Hollódy K. Lamotrigine decreases EEG synchronization in a use-dependent manner in patients with idiopathic generalized epilepsy. Clin Neurophysiol. 2007;118(4):910–7. 17258504 10.1016/j.clinph.2006.11.016

[43] Sannita WG, Gervasio L, Zagnoni P. Quantitative EEG effects and plasma concentration of sodium valproate: acute and long-term administration to epileptic patients. Neuropsychobiology. 1989;22(4):231–5. 2518602 10.1159/000118622

[44] Wu X, Xiao CH. Quantitative pharmaco-EEG of sustained release valproate in epileptics. Clin Electroencephalogr. 1997;28(2):117–20. 9137876 10.1177/155005949702800210

[45] Cho JR, Koo DL, Joo EY, Yoon SM, Ju E, Lee J, et al. Effect of levetiracetam monotherapy on background EEG activity and cognition in drug-naïve epilepsy patients. Clin Neurophysiol. 2012;123(4):664–70. 10.1016/j.clinph.2011.09.012 22000706

