# Supplementary material for: Quantitative analysis of the resting-state EEG power spectrum in patients with epilepsy comorbid with anxiety and depression
Source: Acta Epileptol. 2025 Mar 31;7:22. doi: 10.1186/s42494-025-00206-6 (PMC11956486; doi:10.1186/s42494-025-00206-6)
Supplement: Supplementary file 1 — Supplementary Material 1. [file 42494_2025_206_MOESM1_ESM.docx]

| **Table S1** δ absolute power of each site（×10^12^) | | | |
| --- | --- | --- | --- |
| Sites | Anxiety and depression comorbid in epilepsy（*n*=42） | Absence of epilepsy comorbidity（*n*=45） | *P*-value |
| FP1 | 4.26（5.6） | 5.18（5.65） | 0.677 |
| FP2 | 5.00（6.15） | 5.35（5.98） | 0.993 |
| F3 | 1.65（2.69） | 2.11（3.65） | 0.108 |
| F4 | 1.76（2.6） | 2.32（4.23） | 0.118 |
| C3 | 1.23（1.6） | 1.77（3.28） | 0.094 |
| C4 | 1.33（1.95） | 1.85（3.59） | 0.067 |
| P3 | 1.53（1.89） | 1.74（3.58） | 0.161 |
| P4 | 1.50（1.92） | 1.53（3.57） | 0.234 |
| O1 | 2.58（5.32） | 2.63（6.21） | 0.410 |
| O2 | 2.58（4.48） | 2.76（5.63） | 0.558 |
| F7 | 2.22（2.64） | 2.92（3.22） | 0.209 |
| F8 | 2.41（3.63） | 3.12（3.85） | 0.234 |
| T3 | 1.61（1.58） | 2.34（2.6） | 0.021* |
| T4 | 1.63（1.66） | 2.07（3.17） | 0.124 |
| T5 | 2.14（2.63） | 2.29（4.52） | 0.350 |
| T6 | 1.85（2.05） | 2.08（4.7） | 0.149 |

Note: Data are expressed as median (interquartile range) and were statistically analyzed using the Mann–Whitney U test. * *P* value < 0.05.

| **Table S2** α absolute power of each site（×10^12^) | | | |
| --- | --- | --- | --- |
| Sites | Anxiety and depression comorbid in epilepsy（*n*=42） | Absence of epilepsy comorbidity（*n*=45） | *P*-value |
| FP1 | 2.06（3.81） | 2.95（5.22） | 0.161 |
| FP2 | 2.11（3.75） | 3.13（5.00） | 0.159 |
| F3 | 1.81（3.0） | 2.26（4.02） | 0.122 |
| F4 | 1.73（2.64） | 2.65（4.63） | 0.133 |
| C3 | 1.45（2.31） | 1.79（2.37） | 0.266 |
| C4 | 1.33（2.33） | 1.88（3.07） | 0.142 |
| P3 | 2.18（4.47） | 2.35（7.45） | 0.430 |
| P4 | 2.35（4.67） | 2.60（5.04） | 0.400 |
| O1 | 5.70（9.26） | 5.34 (11.75) | 0.569 |
| O2 | 6.98（11.63） | 9.24 (15.66) | 0.288 |
| F7 | 1.20（2.12） | 2.14 (2.43) | 0.116 |
| F8 | 1.29（2.38） | 2.00 (3.26) | 0.096 |
| T3 | 1.05（1.74） | 1.93 (2.21) | 0.043* |
| T4 | 1.06 (1.65) | 2.10 (2.16) | 0.080 |
| T5 | 3.28 (5.84) | 3.89 (6.21) | 0.368 |
| T6 | 3.20 (3.88) | 3.39 (5.89) | 0.197 |

Note: Data are expressed as median (interquartile range) and were statistically analyzed using the Mann–Whitney U test. * *P* value < 0.05.

| **Table S3** θ relative power of each site（100%） | | | |
| --- | --- | --- | --- |
| Sites | Anxiety and depression comorbid in epilepsy（*n*=42） | Absence of epilepsy comorbidity（*n*=45） | *P*-value |
| Fp1 | 18.23（7.2） | 22.34（10.18） | 0.032* |
| Fp2 | 17.43（7.02） | 19.56（14.24） | 0.064 |
| F3 | 21.39（9.71） | 25.09（12.69） | 0.032* |
| F4 | 22.63（9.4） | 23.27（16.04） | 0.324 |
| C3 | 19.39（6.42） | 21.58（15.47） | 0.096 |
| C4 | 19.1（10.84） | 22.15（12.88） | 0.093 |
| P3 | 18.2（7.41） | 20.18（14.91） | 0.177 |
| P4 | 17.77（10.89） | 19.77（14.3） | 0.292 |
| O1 | 16.68（7.99） | 16.9（14.71） | 0.558 |
| O2 | 15.64（9.56） | 14.38（13.05） | 0.410 |
| F7 | 18.02（6.54） | 21.06（10.47） | 0.047* |
| F8 | 17.97（9.04） | 20.85（12.98） | 0.218 |
| T3 | 19.74（8.5） | 21.17（10.86） | 0.234 |
| T4 | 19.39（7.84） | 21.73（13.84） | 0.105 |
| T5 | 19.74（9.13） | 20.05（18.28） | 0.292 |
| T6 | 19.07（8.91） | 20.52（14.24） | 0.099 |

Note: Data are expressed as median (interquartile range) and were statistically analyzed using the Mann–Whitney U test. * *P* value < 0.05.

To exclude the effect of taking antiseizure medicines on the results of the study, we group the patients according to whether they were taking antiseizure medicines or not, and compare the differences in the EEG power spectrum of the two groups, inclucing 29 cases in the epilepsy unmedicated group and 58 cases in the epilepsy medicated group. The differences of EEG power in each frequency band were compared between the two groups. We found that there was no significant difference in the absolute power value of α band, θ band, δ band and the relative power value of γ band between the two groups (*P* > 0.05). The relative power values of P3, P4, O1, O2 sites in the θ band and P4 site in the β band were statistically different (*P* < 0.05).

**Table S4.1** δ absolute power of each site（×10^12^)

| Sites | Epilepsy without medication（*n*=29） | Epilepsy medication  （*n*=58） | *P*-value |
| --- | --- | --- | --- |
| FP1 | 4.38（3.68） | 4.76（6.97） | 0.979 |
| FP2 | 4.97（4.67） | 5.33（6.67） | 0.741 |
| F3 | 2.14（2.92） | 1.85（3.40） | 0.459 |
| F4 | 2.30（2.57） | 2.03（4.44） | 0.598 |
| C3 | 1.75（1.61） | 1.41（3.69） | 0.844 |
| C4 | 1.76（1.76） | 1.79（2.94） | 0.264 |
| P3 | 1.74（1.88） | 1.60（3.17） | 0.851 |
| P4 | 1.51（1.30） | 1.55（3.39） | 0.605 |
| O1 | 2.63（4.27） | 2.58（5.88） | 0.901 |
| O2 | 2.76（3.68） | 2.76（6.20） | 0.721 |
| F7 | 2.87（1.86） | 2.54（4.17） | 0.809 |
| F8 | 2.97（2.15） | 2.76（5.16） | 0.422 |
| T3 | 1.89（1.17） | 1.89（2.67） | 0.636 |
| T4 | 2.02（1.17） | 1.99（4.19） | 0.416 |
| T5 | 2.24（2.30） | 2.25（5.71） | 0.844 |
| T6 | 1.93（1.89） | 1.91（4.65） | 0.748 |

Note: Data are expressed as median (interquartile range) and were statistically analyzed using the Mann–Whitney U test. * *P* value < 0.05.

**Table S4.2** θ absolute power of each site（×10^12^)

| Sites | Epilepsy without medication（*n*=29） | Epilepsy medication  （*n*=58） | *P*-value |
| --- | --- | --- | --- |
| FP1 | 2.16（1.78） | 1.69（2.64） | 0.428 |
| FP2 | 1.96（1.80） | 1.85（2.74） | 0.685 |
| F3 | 1.51（1.51） | 1.29（2.21） | 0.558 |
| F4 | 1.37（1.93） | 1.54（2.54） | 0.900 |
| C3 | 0.93（1.30） | 0.82（1.49） | 0.633 |
| C4 | 0.93（0.94） | 1.05（1.55） | 0.685 |
| P3 | 1.00（1.29） | 0.96（1.55） | 0.900 |
| P4 | 0.88（1.04） | 0.96（1.73） | 0.719 |
| O1 | 1.85（2.82） | 1.95（3.57） | 0.935 |
| O2 | 2.32（2.50） | 1.98（3.12） | 0.900 |
| F7 | 1.24（0.96） | 1.15（1.87） | 0.943 |
| F8 | 1.14（0.77） | 1.25（2.08） | 0.646 |
| T3 | 1.04（0.92） | 1.04（1.88） | 0.672 |
| T4 | 0.93（0.78） | 1.03（1.72） | 0.428 |
| T5 | 1.73（1.83） | 1.38（2.95） | 0.773 |
| T6 | 1.33（1.25） | 1.50（2.35） | 0.471 |

Note: Data are expressed as median (interquartile range) and were statistically analyzed using the Mann–Whitney U test. * *P* value < 0.05.

**Table S4.3** α absolute power of each site（×10^12^)

| Sites | Epilepsy without medication（*n*=29） | Epilepsy medication  （*n*=58） | *P*-value |
| --- | --- | --- | --- |
| FP1 | 3.05（3.58） | 2.35（4.50） | 0.492 |
| FP2 | 2.94（3.73） | 2.27（4.00） | 0.580 |
| F3 | 2.23（3.01） | 1.80（3.12） | 0.317 |
| F4 | 2.43（3.50） | 1.97（3.73） | 0.486 |
| C3 | 1.79（3.21） | 1.50（2.51） | 0.178 |
| C4 | 1.48（3.26） | 1.56（2.38） | 0.492 |
| P3 | 2.36（9.17） | 2.05（5.00） | 0.391 |
| P4 | 2.62（5.20） | 2.25（4.79） | 0.623 |
| O1 | 6.79（9.71） | 4.37（10.62） | 0.396 |
| O2 | 9.13（17.28） | 5.33（14.14） | 0.442 |
| F7 | 1.92（2.07） | 1.74（2.60） | 0.893 |
| F8 | 1.70（2.75） | 1.64（2.94） | 0.803 |
| T3 | 1.24（2.09） | 1.68（2.15） | 1.000 |
| T4 | 1.40（1.92） | 1.61（2.26） | 0.936 |
| T5 | 4.46（6.68） | 3.24（5.41） | 0.353 |
| T6 | 3.32（3.56） | 3.60（5.45） | 0.986 |

Note: Data are expressed as median (interquartile range) and were statistically analyzed using the Mann–Whitney U test. * *P* value < 0.05.

**Table S4.4** θ relative power of each site（100%）

| Sites | Epilepsy without medication（*n*=29） | Epilepsy medication  （*n*=58） | *P*-value |
| --- | --- | --- | --- |
| FP1 | 0.19（0.10） | 0.20（0.12） | 0.617 |
| FP2 | 0.16（0.10） | 0.18（0.15） | 0.893 |
| F3 | 0.22（0.09） | 0.23（0.13） | 0.377 |
| F4 | 0.23（0.09） | 0.24（0.14） | 0.432 |
| C3 | 0.18（0.07） | 0.21（0.11） | 0.080 |
| C4 | 0.20（0.09） | 0.21（0.13） | 0.120 |
| P3 | 0.17（0.08） | 0.20（0.14） | 0.049* |
| P4 | 0.15（0.09） | 0.20（0.14） | 0.044* |
| O1 | 0.14（0.08） | 0.19（0.13） | 0.022* |
| O2 | 0.13（0.09） | 0.17（0.12） | 0.016* |
| F7 | 0.18（0.06） | 0.20（0.11） | 0.284 |
| F8 | 0.19（0.07） | 0.20（0.11） | 0.326 |
| T3 | 0.20（0.07） | 0.21（0.10） | 0.377 |
| T4 | 0.20（0.08） | 0.20（0.10） | 0.509 |
| T5 | 0.20（0.08） | 0.20（0.16） | 0.257 |
| T6 | 0.19（0.10） | 0.20（0.12） | 0.120 |

Note: Data are expressed as median (interquartile range) and were statistically analyzed using the Mann–Whitney U test. * *P* value < 0.05.

**Table S4.5** β relative power of each site（100%）

| Sites | Epilepsy without medication（*n*=29） | Epilepsy medication  （*n*=58） | *P*-value |
| --- | --- | --- | --- |
| FP1 | 0.03（0.03） | 0.03（0.03） | 0.481 |
| FP2 | 0.03（0.03） | 0.03（0.04） | 0.304 |
| F3 | 0.04（0.04） | 0.04（0.05） | 0.580 |
| F4 | 0.05（0.03） | 0.04（0.04） | 0.427 |
| C3 | 0.04（0.04） | 0.04（0.04） | 0.406 |
| C4 | 0.04（0.04） | 0.04（0.04） | 0.083 |
| P3 | 0.04（0.05） | 0.04（0.04） | 0.131 |
| P4 | 0.05（0.04） | 0.03（0.04） | 0.032* |
| O1 | 0.04（0.05） | 0.04（0.05） | 0.090 |
| O2 | 0.04（0.06） | 0.04（0.04） | 0.246 |
| F7 | 0.05（0.03） | 0.04（0.05） | 0.143 |
| F8 | 0.05（0.03） | 0.04（0.05） | 0.313 |
| T3 | 0.06（0.05） | 0.05（0.06） | 0.116 |
| T4 | 0.06（0.06） | 0.05（0.05） | 0.079 |
| T5 | 0.05（0.04） | 0.04（0.05） | 0.231 |
| T6 | 0.05（0.03） | 0.04（0.04） | 0.097 |

Note: Data are expressed as median (interquartile range) and were statistically analyzed using the Mann–Whitney U test. * *P* value < 0.05.

**STable S4.6** γ relative power of each site（100%）

| Sites | Epilepsy without medication（*n*=29） | Epilepsy medication  （*n*=58） | *P*-value |
| --- | --- | --- | --- |
| FP1 | 0.01（0.01） | 0.01（0.02） | 0.714 |
| FP2 | 0.01（0.01） | 0.01（0.02） | 0.796 |
| F3 | 0.01（0.01） | 0.01（0.02） | 0.950 |
| F4 | 0.01（0.01） | 0.01（0.01） | 0.427 |
| C3 | 0.01（0.01） | 0.01（0.01） | 0.623 |
| C4 | 0.01（0.01） | 0.01（0.01） | 0.809 |
| P3 | 0.01（0.01） | 0.01（0.01） | 0.362 |
| P4 | 0.01（0.01） | 0.01（0.01） | 0.526 |
| O1 | 0.01（0.03） | 0.01（0.03） | 0.406 |
| O2 | 0.01（0.03） | 0.01（0.02） | 1.000 |
| F7 | 0.01（0.01） | 0.02（0.03） | 0.844 |
| F8 | 0.01（0.01） | 0.01（0.03） | 0.993 |
| T3 | 0.02（0.02） | 0.02（0.03） | 0.851 |
| T4 | 0.02（0.02） | 0.02（0.04） | 0.630 |
| T5 | 0.01（0.01） | 0.01（0.01） | 0.411 |
| T6 | 0.01（0.01） | 0.01（0.01） | 0.768 |

Note: Data are expressed as median (interquartile range) and were statistically analyzed using the Mann–Whitney U test. * *P* value < 0.05.

Next, we divided the unmedicated epilepsy group and the medicated epilepsy group into two subgroups according to whether they had comorbid anxiety and depression, and compared the differences in EEG power spectrum between the subgroups. The results showed that there were statistically significant differences in the absolute power spectrum of T3 in the α band, C4 in the δ band, T3 and T4 in the θ band between the patients with and without comorbidities in the drug-free epilepsy group (*P* < 0.05), but no statistically significant differences in the relative power spectrum of each point in the β band and γ band (*P* > 0.05). There were statistically significant differences in the absolute power spectrum of F1 site in the δ band, F3 site in the θ band, C4, O2, T3 site in the β band, and F3, F4, C3, P3, F7, T3 site in the γ band in the epilepsy medication group with or without comorbidities (*P* < 0.05). There was no significant difference in the absolute power spectrum of each point in the α band (*P* > 0.05), and there was no significant difference in the relative power spectrum of each point in the θ band between the two groups with or without comorbidities (*P* > 0.05).

**STable 5.1** δ absolute power of each site（×10^12^)

| Not taking ASMs（*n*=29） taking ASMs（*n*=58） | | | | | | |
| --- | --- | --- | --- | --- | --- | --- |
| sites | Absence of epilepsy comorbidity（*n*=14） | Anxiety and depression comorbid in epilepsy（*n*=15） | *P*-value | Absence of epilepsy comorbidity  （*n*=31） | Anxiety and depression comorbid in epilepsy（*n*=27） | *P*-value |
| Fp1 | 5.31  （3.58） | 4.22  （4.14） | 0.275 | 5.06  （6.93） | 4.41（7.30） | 0.870 |
| Fp2 | 5.43  （4.37） | 4.15  （5.12） | 0.458 | 4.66  （6.26） | 6.13（7.90） | 0.761 |
| F3 | 2.14  （3.40） | 2.33  （2.65） | 1.000 | 2.01  （3.67） | 1.35（2.73） | 0.031* |
| F4 | 2.56  （4.15） | 1.61  （1.99） | 0.163 | 2.21  （4.60） | 1.77（4.28） | 0.265 |
| C3 | 1.86  （1.62） | 1.71  （1.42） | 0.432 | 1.72  （4.90） | 1.22（2.90） | 0.072 |
| C4 | 2.03  （1.59） | 0.99  （1.25） | 0.045* | 1.74  （4.66） | 1.95（2.61） | 0.315 |
| P3 | 2.08  （2.17） | 1.44  （1.68） | 0.163 | 1.61  （6.05） | 1.58（2.83） | 0.346 |
| P4 | 1.54  （1.48） | 1.32  （1.26） | 0.190 | 1.53  （7.56） | 1.57（2.42） | 0.431 |
| O1 | 2.76  （3.51） | 2.57  （5.59） | 0.663 | 2.45  （7.97） | 2.60（5.38） | 0.404 |
| O2 | 2.90  （3.66） | 2.57  （4.06） | 0.407 | 2.54  （7.35） | 2.99（4.55） | 0.623 |
| F7 | 3.07  （1.47） | 2.31  （1.76） | 0.337 | 2.60  （4.41） | 2.14（3.59） | 0.330 |
| F8 | 3.44  （2.07） | 2.13  （2.03） | 0.458 | 2.91  （6.34） | 2.46（3.91） | 0.227 |
| T3 | 2.42  （1.49） | 1.57  （1.04） | 0.011* | 2.01  （3.47） | 1.65（2.65） | 0.163 |
| T4 | 2.17  （1.33） | 1.67  （1.36） | 0.176 | 2.07  （4.70） | 1.44（2.65） | 0.246 |
| T5 | 2.67  （3.06） | 2.21  （1.96） | 0.727 | 2.25  （6.54） | 1.98（4.66） | 0.378 |
| T6 | 2.15  （3.14） | 1.87  （1.39） | 0.295 | 1.94  （6.71） | 1.54（2.61） | 0.215 |

Note: Data are expressed as median (interquartile range) and were statistically analyzed using the Mann–Whitney U test. * *P* value < 0.05.

**STable 5.2** θ absolute power of each site（×10^12^)

| Not taking ASMs（*n*=29） taking ASMs（*n*=58） | | | | | | |
| --- | --- | --- | --- | --- | --- | --- |
| sites | Absence of epilepsy comorbidity  （*n*=14） | Anxiety and depression comorbid in epilepsy（*n*=15） | *P*-value | Absence of epilepsy comorbidity  （*n*=31） | Anxiety and depression comorbid in epilepsy（*n*=27） | *P*-value |
| Fp1 | 2.35  （1.85） | 1.98  （1.77） | 0.206 | 1.90  （5.56） | 1.46（1.79） | 0.233 |
| Fp2 | 2.19  （4.02） | 1.76  （1.79） | 0.239 | 1.94  （4.04） | 1.66（1.63） | 0.370 |
| F3 | 1.67  （3.00） | 1.36  （1.50） | 0.256 | 1.51  （3.13） | 0.89（2.26） | 0.023* |
| F4 | 1.86  （2.80） | 1.01  （1.85） | 0.106 | 1.69  （3.75） | 1.05（2.14） | 0.121 |
| C3 | 0.95  （1.37） | 0.79  （1.17） | 0.315 | 0.95  （2.16） | 0.65（1.61） | 0.097 |
| C4 | 1.02  （1.03） | 0.57  （1.10） | 0.106 | 1.11  （2.84） | 0.90（1.54） | 0.158 |
| P3 | 1.14  （1.41） | 0.76  （1.26） | 0.163 | 1.08  （2.43） | 0.76（1.23） | 0.239 |
| P4 | 1.20  （1.34） | 0.70  （0.83） | 0.097 | 0.93  （2.42） | 0.99（1.33） | 0.300 |
| O1 | 1.84  （3.46） | 1.90  （2.51） | 0.694 | 1.99  （5.53） | 1.81（1.62） | 0.330 |
| O2 | 2.37  （2.96） | 2.14  （2.50） | 0.458 | 2.07  （5.73） | 1.71（2.88） | 0.233 |
| F7 | 1.31  （0.96） | 1.07  （0.88） | 0.315 | 1.52  （3.55） | 0.87（0.92） | 0.060 |
| F8 | 1.21  （0.70） | 1.05  （0.77） | 0.138 | 1.54  （3.30） | 0.84（1.80） | 0.125 |
| T3 | 1.24  （1.05） | 0.74  （0.89） | 0.026* | 1.27  （2.47） | 0.74（1.19） | 0.069 |
| T4 | 1.18  （1.05） | 0.76  （0.51） | 0.026* | 1.14  （1.88） | 0.72（1.76） | 0.121 |
| T5 | 1.76  （3.30） | 1.31  （2.08） | 0.315 | 1.68  （5.24） | 1.19（2.96） | 0.239 |
| T6 | 1.41  （2.48） | 1.29  （0.85） | 0.222 | 1.80  （4.15） | 1.13（1.75） | 0.129 |

Note: Data are expressed as median (interquartile range) and were statistically analyzed using the Mann–Whitney U test. * *P* value < 0.05.

**STable 5.3 α** absolute power of each site（×10^12^)

| Not taking ASMs（*n*=29） taking ASMs（*n*=58） | | | | | | |
| --- | --- | --- | --- | --- | --- | --- |
| sites | Absence of epilepsy comorbidity  （*n*=14） | Anxiety and depression comorbid in epilepsy（*n*=15） | *P*-value | Absence of epilepsy comorbidity  （*n*=31） | Anxiety and depression comorbid in epilepsy（*n*=27） | *P*-value |
| Fp1 | 3.56  （4.96） | 3.05  （3.72） | 0.513 | 2.95  （6.06） | 2.05（2.32） | 0.183 |
| Fp2 | 3.54  （5.23） | 2.95  （3.88） | 0.458 | 3.13  （5.27） | 2.00（2.32） | 0.246 |
| F3 | 2.96  （4.93） | 1.94  （2.86） | 0.359 | 2.14  （3.87） | 1.73（2.80） | 0.188 |
| F4 | 2.90  （5.65） | 1.73  （2.46） | 0.275 | 2.34  （4.24） | 1.73（2.15） | 0.252 |
| C3 | 3.01  （3.84） | 1.43  （2.18） | 0.295 | 1.51  （2.08） | 1.46（2.24） | 0.591 |
| C4 | 2.94  （3.71） | 1.31  （1.28） | 0.206 | 1.60  （2.52） | 1.59（2.48） | 0.378 |
| P3 | 3.14  （9.84） | 2.20  （8.80） | 0.337 | 1.85  （7.43） | 2.17（4.46） | 0.680 |
| P4 | 3.88  （7.47） | 1.87  （4.06） | 0.256 | 2.13  （4.40） | 3.25（4.92） | 0.785 |
| O1 | 7.73  （14.64） | 6.79  （8.32） | 0.600 | 4.36  （10.84） | 4.89（9.99） | 0.612 |
| O2 | 11.38  （17.82） | 8.81（17.24） | 0.570 | 8.20  （15.47） | 4.98（12.01） | 0.286 |
| F7 | 2.73  （2.80） | 1.23  （1.50） | 0.190 | 2.34  （2.20） | 1.16（2.77） | 0.272 |
| F8 | 2.40  （3.14） | 1.27  （2.23） | 0.206 | 1.98  （3.07） | 1.30（2.87） | 0.233 |
| T3 | 2.03  （2.86） | 0.96  （1.09） | 0.045* | 1.89  （2.02） | 1.24（2.15） | 0.286 |
| T4 | 2.10  （2.35） | 0.90  （1.43） | 0.050 | 2.10  （2.25） | 1.29（1.76） | 0.422 |
| T5 | 5.49  （6.91） | 3.40  （6.33） | 0.485 | 3.72  （4.15） | 2.99（6.08） | 0.459 |
| T6 | 3.36  （5.47） | 3.09  （2.76） | 0.275 | 3.88  （5.22） | 3.35（5.01） | 0.396 |

Note: Data are expressed as median (interquartile range) and were statistically analyzed using the Mann–Whitney U test. * *P* value < 0.05.

**STable 5.4** θ relative power of each site（100%）

| Not taking ASMs（*n*=29） taking ASMs（*n*=58） | | | | | | |
| --- | --- | --- | --- | --- | --- | --- |
| sites | Absence of epilepsy comorbidity  （*n*=14） | Anxiety and depression comorbid in epilepsy（*n*=15） | *P*-value | Absence of epilepsy comorbidity  （*n*=31） | Anxiety and depression comorbid in epilepsy（*n*=27） | *P*-value |
| Fp1 | 0.21  （0.09） | 0.18  （0.08） | 0.295 | 0.24  （0.13） | 0.18（0.08） | 0.056 |
| Fp2 | 0.19  （0.11） | 0.19  （0.08） | 0.570 | 0.21  （0.19） | 0.17（0.06） | 0.097 |
| F3 | 0.23  （0.07） | 0.19  （0.09） | 0.138 | 0.26  （0.14） | 0.21（0.11） | 0.137 |
| F4 | 0.23  （0.12） | 0.20  （0.08） | 0.541 | 0.25  （0.18） | 0.24（0.11） | 0.459 |
| C3 | 0.21  （0.08） | 0.19  （0.05） | 0.407 | 0.22  （0.15） | 0.20（0.08） | 0.178 |
| C4 | 0.22  （0.11） | 0.19  （0.08） | 0.631 | 0.22  （0.14） | 0.19（0.10） | 0.088 |
| P3 | 0.16  （0.12） | 0.17  （0.07） | 0.896 | 0.22  （0.16） | 0.19（0.10） | 0.129 |
| P4 | 0.16  （0.09） | 0.15  （0.10） | 0.663 | 0.21  （0.14） | 0.20（0.14） | 0.387 |
| O1 | 0.13  （0.11） | 0.14  （0.08） | 0.631 | 0.21  （0.19） | 0.19（0.11） | 0.450 |
| O2 | 0.13  （0.10） | 0.12  （0.08） | 0.663 | 0.19  （0.17） | 0.17（0.10） | 0.548 |
| F7 | 0.18  （0.06） | 0.16  （0.06） | 0.432 | 0.23  （0.11） | 0.19（0.09） | 0.062 |
| F8 | 0.20  （0.08） | 0.17  （0.07） | 0.383 | 0.21  （0.14） | 0.18（0.10） | 0.404 |
| T3 | 0.19  （0.08） | 0.20  （0.07） | 0.663 | 0.22  （0.12） | 0.20（0.10） | 0.129 |
| T4 | 0.22  （0.08） | 0.19  （0.06） | 0.176 | 0.22  （0.16） | 0.20（0.08） | 0.338 |
| T5 | 0.19  （0.17） | 0.20  （0.06） | 0.930 | 0.20  （0.17） | 0.20（0.12） | 0.279 |
| T6 | 0.20  （0.11） | 0.19  （0.10） | 0.407 | 0.21  （0.15） | 0.20（0.10） | 0.215 |

Note: Data are expressed as median (interquartile range) and were statistically analyzed using the Mann–Whitney U test. * *P* value < 0.05.

**STable 5.5** β relative power of each site（100%）

| Not taking ASMs（*n*=29） taking ASMs（*n*=58） | | | | | | |
| --- | --- | --- | --- | --- | --- | --- |
| sites | Absence of epilepsy comorbidity  （*n*=14） | Anxiety and depression comorbid in epilepsy（*n*=15） | *P*-value | Absence of epilepsy comorbidity  （*n*=31） | Anxiety and depression comorbid in epilepsy（*n*=27） | *P*-value |
| Fp1 | 0.25  （0.02） | 0.03  （0.04） | 0.163 | 0.03  （0.03） | 0.04（0.03） | 0.498 |
| Fp2 | 0.03  （0.02） | 0.03  （0.05） | 0.827 | 0.03  （0.04） | 0.04（0.03） | 0.498 |
| F3 | 0.04  （0.03） | 0.04  （0.04） | 0.663 | 0.03  （0.05） | 0.05（0.05） | 0.107 |
| F4 | 0.04  （0.03） | 0.05  （0.04） | 0.407 | 0.03  （0.03） | 0.06（0.05） | 0.103 |
| C3 | 0.04  （0.04） | 0.04  （0.05） | 0.432 | 0.03  （0.04） | 0.05（0.04） | 0.052 |
| C4 | 0.04  （0.03） | 0.05  （0.05） | 0.116 | 0.03  （0.03） | 0.04（0.04） | 0.045* |
| P3 | 0.04  （0.04） | 0.05  （0.05） | 0.337 | 0.03  （0.04） | 0.04（0.05） | 0.079 |
| P4 | 0.04  （0.04） | 0.05  （0.05） | 0.190 | 0.03  （0.04） | 0.03（0.04） | 0.141 |
| O1 | 0.04  （0.06） | 0.05  （0.05） | 0.930 | 0.03  （0.03） | 0.04（0.05） | 0.072 |
| O2 | 0.03  （0.04） | 0.04  （0.06） | 0.383 | 0.03  （0.03） | 0.05（0.04） | 0.049* |
| F7 | 0.04  （0.03） | 0.06  （0.05） | 0.432 | 0.03  （0.04） | 0.05（0.04） | 0.082 |
| F8 | 0.03  （0.02） | 0.05  （0.03） | 0.127 | 0.03  （0.04） | 0.05（0.06） | 0.239 |
| T3 | 0.06  （0.04） | 0.06  （0.07） | 0.485 | 0.03  （0.04） | 0.06（0.05） | 0.021* |
| T4 | 0.06  （0.06） | 0.06  （0.06） | 0.337 | 0.03  （0.04） | 0.06（0.06） | 0.154 |
| T5 | 0.04  （0.04） | 0.05  （0.03） | 0.359 | 0.03  （0.03） | 0.04（0.05） | 0.067 |
| T6 | 0.04  （0.03） | 0.05  （0.05） | 0.127 | 0.03  （0.04） | 0.04（0.05） | 0.082 |

Note: Data are expressed as median (interquartile range) and were statistically analyzed using the Mann–Whitney U test. * *P* value < 0.05.

**STable 5.6** γ relative power of each site（100%）

| Not taking ASMs（*n*=29） taking ASMs（*n*=58） | | | | | | |
| --- | --- | --- | --- | --- | --- | --- |
| sites | Absence of epilepsy comorbidity  （*n*=14） | Anxiety and depression comorbid in epilepsy（*n*=15） | *P*-value | Absence of epilepsy comorbidity  （*n*=31） | Anxiety and depression comorbid in epilepsy（*n*=27） | *P*-value |
| Fp1 | 0.01  （0.01） | 0.01  （0.01） | 0.485 | 0.01  （0.02） | 0.02（0.02） | 0.459 |
| Fp2 | 0.01  （0.01） | 0.01  （0.00） | 0.965 | 0.01  （0.02） | 0.01（0.02） | 0.145 |
| F3 | 0.01  （0.01） | 0.01  （0.01） | 0.760 | 0.01  （0.01） | 0.01（0.02） | 0.027* |
| F4 | 0.01  （0.02） | 0.01  （0.01） | 0.600 | 0.01  （0.01） | 0.01（0.01） | 0.037* |
| C3 | 0.01  （0.01） | 0.01  （0.01） | 0.541 | 0.01  （0.01） | 0.01（0.02） | 0.042* |
| C4 | 0.01  （0.01） | 0.01  （0.01） | 0.383 | 0.01  （0.01） | 0.01（0.01） | 0.062 |
| P3 | 0.01  （0.01） | 0.01  （0.01） | 0.541 | 0.01  （0.01） | 0.01（0.01） | 0.035* |
| P4 | 0.01  （0.01） | 0.01  （0.01） | 0.407 | 0.01  （0.01） | 0.01（0.01） | 0.117 |
| O1 | 0.01  （0.03） | 0.01  （0.04） | 0.965 | 0.01  （0.02） | 0.01（0.03） | 0.121 |
| O2 | 0.01  （0.03） | 0.01  （0.02） | 0.485 | 0.01  （0.01） | 0.01（0.04） | 0.137 |
| F7 | 0.02  （0.01） | 0.02  （0.02） | 0.359 | 0.01  （0.02） | 0.02（0.02） | 0.035* |
| F8 | 0.01  （0.01） | 0.02  （0.02） | 0.081 | 0.01  （0.03） | 0.02（0.03） | 0.168 |
| T3 | 0.02  （0.03） | 0.02  （0.02） | 0.570 | 0.01  （0.02） | 0.03（0.05） | 0.005* |
| T4 | 0.02  （0.03） | 0.02  （0.02） | 0.432 | 0.02  （0.02） | 0.02（0.07） | 0.422 |
| T5 | 0.01  （0.01） | 0.01  （0.01） | 0.337 | 0.01  （0.01） | 0.01（0.03） | 0.058 |
| T6 | 0.01  （0.01） | 0.01  （0.01） | 0.432 | 0.01  （0.01） | 0.01（0.01） | 0.121 |

Note: Data are expressed as median (interquartile range) and were statistically analyzed using the Mann–Whitney U test. * *P* value < 0.05.

Finally, the epilepsy comorbidity with anxiety and depression group and the epilepsy without comorbidity group were further divided into two subgroups according to whether they took anti-seizure drugs or not, and the differences in EEG power spectrum between the two subgroups were compared. There was no significant difference in the absolute power value of α, θ, δ frequency band and the relative power value of θ, β, γ frequency band between the epilepsy comorbidity with anxiety and depression group and the epilepsy without comorbidity group (p > 0.05).

**STable 6.1** δ absolute power of each site（×10^12^)

| Anxiety and depression comorbid in epilepsy（*n*=42）Absence of epilepsy comorbidity（*n*=45） | | | | | | |
| --- | --- | --- | --- | --- | --- | --- |
| sites | Not taking ASMs  （*n*=15） | taking ASMs  （*n*=27） | *P*-value | Not taking ASMs  （*n*=14） | taking ASMs  （*n*=31） | *P*-value |
| Fp1 | 4.23  （4.14） | 4.41  （7.30） | 0.844 | 5.31  （3.58） | 5.06（6.93） | 0.750 |
| Fp2 | 4.15  （5.12） | 6.13  （7.90） | 0.590 | 5.43  （4.37） | 4.66（6.26） | 0.607 |
| F3 | 2.33  （2.65） | 1.35  （2.73） | 0.131 | 2.14  （3.40） | 2.01（3.67） | 0.941 |
| F4 | 1.61  （1.99） | 1.77  （4.28） | 0.743 | 2.56  （4.15） | 2.21（4.60） | 0.864 |
| C3 | 1.71  （1.42） | 1.22  （2.90） | 0.365 | 1.86  （1.62） | 1.72（4.90） | 0.941 |
| C4 | 0.99  （1.25） | 1.95  （2.61） | 0.312 | 2.03  （1.59） | 1.74（4.66） | 0.825 |
| P3 | 1.44  （1.68） | 1.58  （2.83） | 0.906 | 2.76  （2.17） | 1.61（6.05） | 0.659 |
| P4 | 1.32  （1.26） | 1.57  （2.42） | 0.646 | 1.54  （1.48） | 1.53（7.56） | 0.787 |
| O1 | 2.57  （5.59） | 2.60  （5.38） | 0.590 | 2.76  （3.51） | 2.45（7.97） | 0.845 |
| O2 | 2.57  （4.06） | 2.99  （4.55） | 0.743 | 2.90  （3.66） | 2.54（7.35） | 0.492 |
| F7 | 2.31  （1.76） | 2.14  （3.59） | 0.684 | 3.07  （1.47） | 2.60（4.41） | 1.000 |
| F8 | 2.13  （2.03） | 2.46  （3.91） | 0.906 | 3.44  （2.07） | 2.91（6.34） | 0.787 |
| T3 | 1.57  （1.04） | 1.65  （2.65） | 0.865 | 2.42  （1.49） | 2.01（3.47） | 0.695 |
| T4 | 1.64  （1.36） | 1.44  （2.65） | 0.723 | 2.17  （1.33） | 2.07（4.70） | 0.806 |
| T5 | 2.21  （1.96） | 1.98  （4.66） | 0.487 | 2.67  （3.06） | 2.25（6.54） | 0.845 |
| T6 | 1.87  （1.39） | 1.54  （2.61） | 0.969 | 2.15  （3.14） | 1.94（6.71） | 0.922 |

Note: Data are expressed as median (interquartile range) and were statistically analyzed using the Mann–Whitney U test. * *P* value < 0.05.

**STable 6.2** θ absolute power of each site（×10^12^)

| Anxiety and depression comorbid in epilepsy（*n*=42）Absence of epilepsy comorbidity（*n*=45） | | | | | | |
| --- | --- | --- | --- | --- | --- | --- |
| sites | Not taking ASMs  （*n*=15） | taking ASMs  （*n*=27） | *P*-value | Not taking ASMs  （*n*=14） | taking ASMs  （*n*=31） | *P*-value |
| Fp1 | 1.98  （1.77） | 1.46  （1.79） | 0.487 | 2.35  （1.85） | 1.90（5.56） | 0.624 |
| Fp2 | 1.76  （1.79） | 1.66  （1.63） | 0.763 | 2.19  （4.02） | 1.94（4.04） | 0.641 |
| F3 | 1.36  （1.50） | 0.89  （2.26） | 0.365 | 1.67  （3.00） | 1.51（3.13） | 0.806 |
| F4 | 1.01  （1.85） | 1.05  （2.14） | 0.990 | 1.86  （2.80） | 1.69（3.75） | 0.845 |
| C3 | 0.79  （1.17） | 0.65  （1.61） | 0.503 | 0.95  （1.37） | 0.95（2.16） | 0.806 |
| C4 | 0.57  （1.10） | 0.90  （1.54） | 0.885 | 1.02  （1.03） | 1.11（2.84） | 1.000 |
| P3 | 0.76  （1.26） | 0.76  （1.23） | 0.990 | 1.14  （1.41） | 1.08（2.43） | 0.695 |
| P4 | 0.70  （0.83） | 0.99  （1.33） | 0.665 | 1.20  （1.34） | 0.93（2.42） | 1.000 |
| O1 | 1.90  （2.51） | 1.81  （1.62） | 0.803 | 1.84  （3.46） | 1.99（5.53） | 0.845 |
| O2 | 2.14  （2.50） | 1.71  （2.88） | 0.906 | 2.37  （2.96） | 2.07（5.73） | 0.750 |
| F7 | 1.07  （0.88） | 0.87  （0.92） | 0.627 | 1.31  （0.96） | 1.52（3.55） | 0.695 |
| F8 | 1.05  （0.77） | 0.83  （1.80） | 0.948 | 1.21  （0.70） | 1.54（3.30） | 0.659 |
| T3 | 0.74  （0.89） | 0.74  （1.19） | 0.865 | 1.24  （1.05） | 1.27（2.47） | 0.961 |
| T4 | 0.76  （0.51） | 0.72  （1.76） | 0.470 | 1.18  （1.05） | 1.14（1.88） | 0.902 |
| T5 | 1.31  （2.08） | 1.19  （2.96） | 0.763 | 1.76  （3.30） | 1.68（5.24） | 0.713 |
| T6 | 1.29  （0.85） | 1.13  （1.75） | 0.609 | 1.41  （2.48） | 1.80（4.15） | 0.677 |

Note: Data are expressed as median (interquartile range) and were statistically analyzed using the Mann–Whitney U test. **P* value < 0.05.

**STable 6.3 α** absolute power of each site（×10^12^)

| Anxiety and depression comorbid in epilepsy（*n*=42）Absence of epilepsy comorbidity（*n*=45） | | | | | | |
| --- | --- | --- | --- | --- | --- | --- |
| sites | Not taking ASMs  （*n*=15） | taking ASMs  （*n*=27） | *P*-value | Not taking ASMs  （*n*=14） | taking ASMs  （*n*=31） | *P*-value |
| Fp1 | 3.05  （3.72） | 2.05  （2.32） | 0.338 | 3.57  （4.96） | 2.95（6.06） | 0.508 |
| Fp2 | 2.95  （3.88） | 2.00  （2.32） | 0.423 | 3.55  （5.23） | 3.13（5.27） | 0.556 |
| F3 | 1.94  （2.86） | 1.73  （2.80） | 0.265 | 1.96  （4.93） | 2.14（3.87） | 0.292 |
| F4 | 1.72  （2.46） | 1.73  （2.15） | 0.423 | 2.90  （5.65） | 2.34（4.24） | 0.405 |
| C3 | 1.43  （2.18） | 1.46  （2.24） | 0.503 | 3.01  （3.84） | 1.51（2.08） | 0.062 |
| C4 | 1.31  （1.28） | 1.56  （2.48） | 0.783 | 2.94  （3.71） | 1.60（2.52） | 0.178 |
| P3 | 2.20  （8.80） | 2.17  （4.46） | 0.627 | 3.14  （9.84） | 1.85（7.43） | 0.270 |
| P4 | 1.87  （4.06） | 3.25  （4.92） | 0.969 | 3.88  （7.47） | 2.13（4.40） | 0.230 |
| O1 | 6.79  （8.32） | 4.89  （9.99） | 0.379 | 7.43  （14.64） | 4.36（10.84） | 0.405 |
| O2 | 8.81  （17.24） | 4.98（12.01） | 0.338 | 11.38  （17.82） | 8.20（15.47） | 0.462 |
| F7 | 1.23  （1.50） | 1.56  （2.77） | 0.763 | 2.73  （2.80） | 2.34（2.20） | 0.462 |
| F8 | 1.27  （2.23） | 1.30  （2.87） | 0.969 | 2.40  （3.14） | 1.96（3.07） | 0.540 |
| T3 | 0.96  （1.09） | 1.24  （2.15） | 0.803 | 2.03  （2.86） | 1.89（2.02） | 0.327 |
| T4 | 0.90  （1.43） | 1.29  （1.76） | 0.646 | 2.10  （2.35） | 2.10（2.25） | 0.352 |
| T5 | 3.40  （6.33） | 2.99  （6.08） | 0.470 | 5.46  （6.91） | 3.72（4.15） | 0.230 |
| T6 | 3.09  （2.76） | 3.35  （5.01） | 0.990 | 3.56  （5.47） | 3.88（5.22） | 0.573 |

Note: Data are expressed as median (interquartile range) and were statistically analyzed using the Mann–Whitney U test. **P* value < 0.05.

**STable 6.4** θ relative power of each site（100%）

| Anxiety and depression comorbid in epilepsy（*n*=42）Absence of epilepsy comorbidity（*n*=45） | | | | | | |
| --- | --- | --- | --- | --- | --- | --- |
| sites | Not taking ASMs  （*n*=15） | taking ASMs  （*n*=27） | p-value | Not taking ASMs  （*n*=14） | taking ASMs  （*n*=31） | *P*-value |
| Fp1 | 0.18  （0.08） | 0.18  （0.08） | 0.865 | 0.21  （0.09） | 0.24（0.13） | 0.573 |
| Fp2 | 0.16  （0.08） | 0.17  （0.06） | 0.423 | 0.19  （0.11） | 0.21（0.19） | 0.659 |
| F3 | 0.19  （0.09） | 0.21  （0.11） | 0.743 | 0.23  （0.07） | 0.26（0.14） | 0.713 |
| F4 | 0.20  （0.08） | 0.24  （0.11） | 0.555 | 0.23  （0.12） | 0.25（0.18） | 0.750 |
| C3 | 0.19  （0.05） | 0.20  （0.08） | 0.312 | 0.21  （0.08） | 0.22（0.15） | 0.178 |
| C4 | 0.19  （0.08） | 0.19  （0.10） | 0.487 | 0.22  （0.11） | 0.22（0.14） | 0.249 |
| P3 | 0.17  （0.07） | 0.19  （0.10） | 0.439 | 0.16  （0.12） | 0.21（0.16） | 0.111 |
| P4 | 0.15  （0.10） | 0.20  （0.14） | 0.265 | 0.16  （0.09） | 0.21（0.14） | 0.155 |
| O1 | 0.14  （0.08） | 0.19  （0.11） | 0.112 | 0.13  （0.11） | 0.21（0.19） | 0.111 |
| O2 | 0.12  （0.08） | 0.17  （0.10） | 0.090 | 0.13  （0.10） | 0.19（0.17） | 0.100 |
| F7 | 0.16  （0.06） | 0.19  （0.09） | 0.803 | 0.18  （0.06） | 0.23（0.11） | 0.239 |
| F8 | 0.17  （0.07） | 0.18  （0.10） | 0.665 | 0.20  （0.08） | 0.21（0.14） | 0.607 |
| T3 | 0.20  （0.07） | 0.20  （0.10） | 0.609 | 0.19  （0.08） | 0.22（0.12） | 0.155 |
| T4 | 0.19  （0.06） | 0.20  （0.08） | 0.627 | 0.22  （0.08） | 0.22（0.16） | 0.922 |
| T5 | 0.20  （0.06） | 0.20  （0.12） | 0.783 | 0.19  （0.17） | 0.20（0.17） | 0.418 |
| T6 | 0.19  （0.10） | 0.20  （0.10） | 0.312 | 0.20  （0.11） | 0.21（0.15） | 0.433 |

Note: Data are expressed as median (interquartile range) and were statistically analyzed using the Mann–Whitney U test. * *P* value < 0.05.

**STable 6.5** β relative power of each site（100%）

| Anxiety and depression comorbid in epilepsy（*n*=42）Absence of epilepsy comorbidity（*n*=45） | | | | | | |
| --- | --- | --- | --- | --- | --- | --- |
| sites | Not taking ASMs  （*n*=15） | taking ASMs  （*n*=27） | *P*-value | Not taking ASMs  （*n*=14） | taking ASMs  （*n*=31） | *P*-value |
| Fp1 | 0.03  （0.04） | 0.35  （0.03） | 0.423 | 0.02  （0.02） | 0.03（0.03） | 0.922 |
| Fp2 | 0.03  （0.05） | 0.04  （0.03） | 0.555 | 0.03  （0.02） | 0.03（0.04） | 0.573 |
| F3 | 0.04  （0.04） | 0.05  （0.05） | 0.803 | 0.04  （0.03） | 0.03（0.05） | 0.391 |
| F4 | 0.05  （0.04） | 0.06  （0.05） | 0.763 | 0.04  （0.03） | 0.03（0.03） | 0.405 |
| C3 | 0.04  （0.05） | 0.05  （0.04） | 0.969 | 0.04  （0.04） | 0.03（0.04） | 0.433 |
| C4 | 0.05  （0.05） | 0.04  （0.04） | 0.365 | 0.04  （0.03） | 0.03（0.03） | 0.327 |
| P3 | 0.05  （0.05） | 0.04  （0.05） | 0.555 | 0.04  （0.04） | 0.03（0.04） | 0.391 |
| P4 | 0.05  （0.05） | 0.03  （0.04） | 0.168 | 0.04  （0.04） | 0.03（0.04） | 0.249 |
| O1 | 0.05  （0.05） | 0.04  （0.05） | 0.823 | 0.04  （0.06） | 0.03（0.03） | 0.117 |
| O2 | 0.04  （0.06） | 0.05  （0.04） | 0.590 | 0.03  （0.04） | 0.03（0.03） | 0.524 |
| F7 | 0.06  （0.05） | 0.05  （0.04） | 0.723 | 0.04  （0.03） | 0.03（0.04） | 0.178 |
| F8 | 0.05  （0.03） | 0.05  （0.06） | 0.627 | 0.03  （0.02） | 0.03（0.04） | 0.508 |
| T3 | 0.06  （0.07） | 0.06  （0.05） | 0.990 | 0.06  （0.04） | 0.03（0.04） | 0.141 |
| T4 | 0.06  （0.06） | 0.06  （0.06） | 0.537 | 0.06  （0.06） | 0.03（0.04） | 0.303 |
| T5 | 0.05  （0.03） | 0.04  （0.05） | 0.927 | 0.04  （0.04） | 0.03（0.03） | 0.377 |
| T6 | 0.05  （0.05） | 0.04  （0.05） | 0.194 | 0.04  （0.03） | 0.03（0.04） | 0.556 |

Note: Data are expressed as median (interquartile range) and were statistically analyzed using the Mann–Whitney U test. * *P* value < 0.05.

**STable 6.6** γ relative power of each site（100%）

| Anxiety and depression comorbid in epilepsy（*n*=42)Absence of epilepsy comorbidity（*n*=45） | | | | | | |
| --- | --- | --- | --- | --- | --- | --- |
| sites | Not taking ASMs  （*n*=15） | taking ASMs  （*n*=27） | *P*-value | Not taking ASMs  （*n*=14） | taking ASMs  （*n*=31） | *P*-value |
| Fp1 | 0.01  （0.01） | 0.02  （0.02） | 0.537 | 0.01  （0.01） | 0.01（0.02） | 0.825 |
| Fp2 | 0.01  （0.00） | 0.01  （0.02） | 0.503 | 0.01  （0.01） | 0.01（0.02） | 0.477 |
| F3 | 0.01  （0.01） | 0.01  （0.02） | 0.203 | 0.01  （0.01） | 0.01（0.01） | 0.556 |
| F4 | 0.01  （0.01） | 0.01  （0.01） | 0.703 | 0.01  （0.02） | 0.01（0.01） | 0.405 |
| C3 | 0.01  （0.01） | 0.01  （0.02） | 0.265 | 0.01  （0.01） | 0.01（0.01） | 0.961 |
| C4 | 0.01  （0.01） | 0.01  （0.01） | 0.572 | 0.01  （0.01） | 0.01（0.01） | 0.750 |
| P3 | 0.01  （0.01） | 0.01  （0.01） | 0.646 | 0.01  （0.01） | 0.01（0.01） | 0.327 |
| P4 | 0.01  （0.01） | 0.01  （0.01） | 0.969 | 0.01  （0.01） | 0.01（0.01） | 0.624 |
| O1 | 0.01  （0.04） | 0.01  （0.03） | 0.665 | 0.01  （0.03） | 0.01（0.02） | 0.303 |
| O2 | 0.01  （0.02） | 0.01  （0.04） | 0.743 | 0.01  （0.03） | 0.01（0.01） | 0.825 |
| F7 | 0.01  （0.02） | 0.02  （0.02） | 0.423 | 0.02  （0.01） | 0.01（0.02） | 0.540 |
| F8 | 0.02  （0.02） | 0.02  （0.03） | 0.906 | 0.01  （0.01） | 0.01（0.03） | 0.677 |
| T3 | 0.02  （0.02） | 0.03  （0.05） | 0.081 | 0.02  （0.03） | 0.01（0.02） | 0.239 |
| T4 | 0.02  （0.02） | 0.02  （0.07） | 0.783 | 0.02  （0.03） | 0.02（0.02） | 0.961 |
| T5 | 0.01  （0.01） | 0.01  （0.03） | 0.723 | 0.01  （0.01） | 0.01（0.01） | 0.418 |
| T6 | 0.01  （0.01） | 0.01  （0.01） | 0.743 | 0.01  （0.01） | 0.01（0.01） | 0.902 |

Note: Data are expressed as median (interquartile range) and were statistically analyzed using the Mann–Whitney U test. * *P* value < 0.05.
